# Midday Nap Duration and Hypertension among Middle-Aged and Older Chinese Adults: A Nationwide Retrospective Cohort Study

**DOI:** 10.3390/ijerph18073680

**Published:** 2021-04-01

**Authors:** Jialin Fu, Xinge Zhang, Justin B. Moore, Bowen Wang, Rui Li

**Affiliations:** 1 Department of Preventive Medicine, School of Health Sciences, Wuhan University, Wuhan 430071, China; Fjl0708@whu.edu.cn (J.F.); wbw9630@whu.edu.cn (B.W.); 2 Department of Medicine and Therapeutics, The Chinese University of Hong Kong, Prince of Wales Hospital, Hong Kong, China; xinge@link.cuhk.edu.hk; 3 Department of Implementation Science, Wake Forest School of Medicine, Medical Center Boulevard, Winston-Salem, NC 27157, USA; jusmoore@wakehealth.edu; 4 Department of Epidemiology & Prevention, Wake Forest School of Medicine, Medical Center Boulevard, Winston-Salem, NC 27157, USA; 5 Department of Health Management, School of Health Sciences, Wuhan University, Wuhan 430071, China

**Keywords:** midday nap duration, hypertension, Chinese adults

## Abstract

The goal of this study was to investigate the associations of midday nap duration and change in midday nap duration with hypertension in a retrospective cohort using a nationwide representative sample of middle-aged and older Chinese adults. Data were obtained from the China Health and Retirement Longitudinal Study (CHARLS) database during 2011–2015. Information on midday nap duration was collected via a self-reported questionnaire and blood pressure was objectively measured. Hazard ratios (HR) with 95% confidence interval (CI) were estimated using Cox proportional hazards regression models to quantify the associations. A sample of 5729 Chinese adults (≥45 years old) were included in the longitudinal analysis. Relative to non-nappers, participants who napping for ≥90 min/day was associated with significantly larger HR for hypertension at four-year follow-up (HR = 1.18, 95% CI = 1.01–1.40, *p* = 0.048). Compared with people who napped ≥90 min/day both at baseline (2011) and follow-up (2013), hypertension risk at four-year follow-up declined in individuals whose midday nap durations decreased in the 2-year study period from ≥ 90 min/day to 1–59 min/day (HR = 0.59, 95% CI = 0.36–0.97, *p* = 0.037) and 60–89 min/day (HR = 0.68, 95% CI = 0.47–0.99, *p* = 0.044). Among middle-aged and older Chinese adults, relative to non-nappers, people who had longer midday nap duration (≥90 min/day) were associated with significantly larger HR for hypertension and decreased napping duration may confer benefit for hypertension prevention.

## 1. Introduction

High blood pressure is one of the most important risks for morbidity and mortality [1]. Many studies have identified high blood pressure as the best example of a surrogate measure for cardiovascular diseases, especially for stroke [2,3,4]. Hypertension, characterized by chronically elevated blood pressure above 140/90 mmHg, is one of the strongest risk factors of common cardiovascular diseases [5]. According to a study involving 200 countries, the number of adults with elevated blood pressure increased from 594 million in 1975 to 1.13 billion in 2015, comprising 597 million men and 529 million women [6]. High systolic blood pressure (SBP) was the leading risk factor of global disability-adjusted life-years (DALYs), and the cause of 10.4 million deaths and 218 million DALYs in 2017 [7]. In China, a nationwide survey completed in 2017 estimated that nearly half of the Chinese adults aged 35–75 years had hypertension [8]. Numerous studies have shown that age, gender, race/ethnicity [9], and lifestyle factors such as physical inactivity [10], high salt intake [5], obesity [11], and sleep duration [12] are risk factors for hypertension.

Napping is short sleep, typically taken during daylight hours. It is a prevalent lifestyle practice around the world, including China [13]. Some studies suggesting napping is beneficial to health by improving somatic functioning and working efficiency in the daytime [14] and counteracting sleep disorders (e.g., insomnia and sleep apnea) [15]. However, a growing number of studies reported adverse outcomes of daytime napping, such as increasing the odds of cardiovascular disease and all-cause mortality [16]. Daytime napping has been found associated with higher levels of inflammatory markers, such as C-reactive protein, in older adults [17]. As such, long daytime napping might increase risks of cardiovascular disease and all-cause mortality via chronic low-grade inflammation.

Previous studies have indicated the association between midday nap duration and hypertension, but the results were not consistent. Several studies have found that midday nap is associated with a greater risk of hypertension [18,19,20,21,22,23,24], while other studies have shown lower risk [25,26,27,28,29]. Currently, the existing literature regarding this issue lacks a high-quality prospective cohort study in China. More importantly, no study has yet examined the association between changes in nap duration and hypertension, which is important since sleep is a dynamic process over a lifetime [30]. Most epidemiological studies employed a single assessment of sleep information at one time, which limits the strength of observing any correlational relationships regarding sleep time change [31].

Thus, based on the CHARLS, a nationwide retrospective cohort study, we aimed to investigate the longitudinal association between midday naptime and hypertension among middle-aged and older Chinese adults.

## 2. Materials and Methods

### 2.1. Study Participants

Our research used data from the CHARLS, which was publicly available at http://charls.pku.edu.cn (accessed on 30 March 2021). CHARLS was a national representative investigation among 45-year-old or older Chinese adults. Participants from 450 villages or communities in 150 districts of 28 participating provinces were recruited using a stratified multistage probability-proportional-to-size random-cluster sampling strategy. The baseline survey was conducted to assess the social, economic, and health circumstances of community-residents in 2011 with a response rate of 80.5%. Respondents were periodically re-surveyed every two years using a face-to-face computer-assisted personal interview. The detailed methodology description and core questionnaire of the CHARLS have been described elsewhere [32]. The data used in this research were from the CHARLS 2011 baseline survey and follow-up in 2013 and 2015 and analyses were performed in 2020.

**Figure 1:**
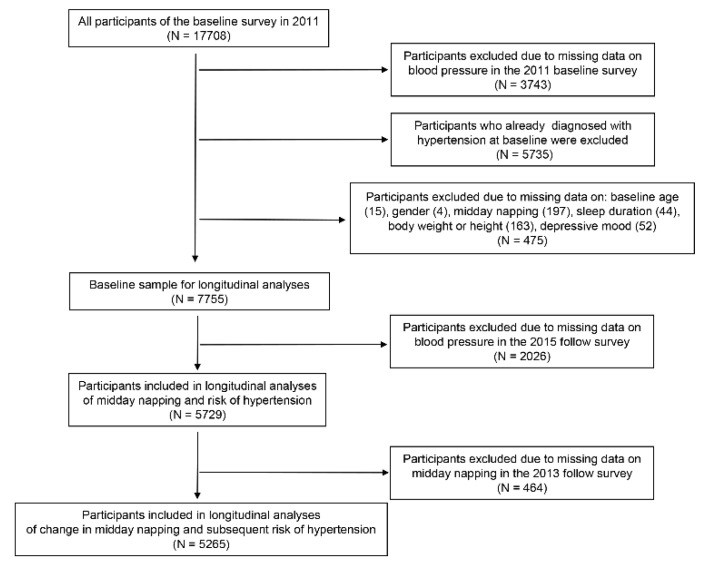
Flow chart of participants.

Out of a total of 17,708 respondents at baseline, 3743 subjects were excluded due to lack of data on blood pressure in the 2011 baseline survey, and 475 subjects were excluded due to missing data on baseline age, gender, height, weight, sleep duration, and midday napping in the 2011 baseline survey. An additional 5735 participants already diagnosed with hypertension at baseline were excluded from the longitudinal analysis. Similarly, 2026 subjects were excluded due to missing data on blood pressure at the four-year follow-up in 2015, resulting in a sample of 5729 adults who were included in the analysis of midday nap duration and the incident hypertension. We utilized data from this subsample of participants with complete data on midday napping with at least two visits (2011 and 2013) to assess the association between change in midday nap duration and hypertension risk in the subsequent 4 years (until the end of 2015). On the basis of the sample of analyzing the longitudinal association between midday nap duration and the incident hypertension, we excluded 464 subjects with missing data on midday napping in 2013, 5265 subjects were included in the analysis. (Figure 1)

### 2.2. Measurements of Sleep Duration and Hypertension

Midday nap duration was appraised using a self-reported questionnaire which asked, “During the past month, how long did you take a nap after lunch on average?” Sleep duration was assessed by the following question: “During the past month, how many hours of actual sleep did you get at night (average hours for one night)? This may be shorter than the number of hours you spend in bed.” These two questions had high validity and reliability for measuring the length of midday nap and night sleep [33,34].

Diastolic blood pressure (DBP) and SBP levels were measured using a mercury sphygmomanometer. Hypertension was diagnosed if an individual satisfied any of the four criteria: SBP ≥ 140 mmHg, DBP ≥ 90 mmHg, self-report of a diagnosis of hypertension, or currently taking antihypertensive medication [35]. Baseline hypertension was available from the baseline survey in 2011, and incident hypertension was defined as participants who were classified as having hypertension in either follow-up wave at four-year follow-up.

### 2.3. Potential Confounders

A structured questionnaire was used to collect information on sociodemographic characteristics (age, gender), and health-related variables including smoking status (current = 2, former = 1, or never = 0), drinking status (current = 2, former = 1, or never = 0), depression (yes = 1 or no = 0), and personal medical histories (hyperlipidemia and diabetes). BMI was objectively measured, calculated as the body weight in kilograms divided by height in meters squared (kg/m^2^). Depressive symptoms were assessed using the 10-item Center for Epidemiological Studies Depression Scale (CES-D-10), which has 10 questions with a scale of four points, and a cutoff score ≥ 10 was used to identify the respondents who had significant depressive symptoms [36]. Personal medical histories were appraised using the following questions: “Have you been diagnosed with hyperlipidemia by a doctor?” and “Have you been diagnosed with diabetes by a doctor?”.

### 2.4. Statistical Analyses

Baseline characteristics of participants were reported in strata of midday nap duration groups. Normality (Shapiro–Wilk test) and variances (Levene’s test) were performed. Data were compared using analysis of variance tests or Kruskal and Wallis test for continuous variables and χ^2^ analysis for categorical variables. Person-years for each subject were calculated from the date of recruitment until the date of the first hypertension event or the follow-up survey in 2015, whichever came first.

HR with 95% CI was estimated using Cox proportional hazards regression models with follow-up period as the time scale to assess the associations of baseline midday nap duration and changes in midday nap durations over two years with subsequent risks of hypertension. Age, gender, BMI, smoking status, drinking status, sleep duration, depressive mood, and personal medical histories (hyperlipidemia and diabetes) at baseline were included as potential confounders. For the purpose of analyzing the longitudinal association between midday nap duration and hypertension, following previous works, [22,24] we created five categories (0 min, 1–29 min, 30–59 min, 60–89 min, ≥90 min) according to the baseline (2011) midday napping, with the group who napped 0 min/day used as the reference group.

We used the changes of midday napping from baseline in 2011 to the first follow-up in 2013, as the exposure to evaluate the potential influence of change in midday nap duration on hypertension. As only 114 hypertension cases occurred in participants who had a midday nap less than 30 min, and 109 hypertension cases occurred in subjects who reported having a midday nap of 30–59 min, we combined 1–29 min and 30–59 min into 1–59 min, and divided midday napping into four groups (0 min, 1–59 min, 60–89 min, ≥90 min). The change in midday nap duration between the baseline survey (2011) and the first follow-up survey (2013) was coded through combinations of the four groups. Sixteen categories of change in midday nap duration were constructed and groups with the same midday nap duration categories at baseline (2011) and follow-up (2013) were used as the reference groups in our analysis.

All statistical analyses were performed using Stata version 16.0 (Stata Corp, College Station, TX, USA). All *p*-values were two-tailed, and *p*-values < 0.05 were considered statistically significant.

## 3. Results

Of 13,965 subjects with blood pressure at baseline, 5735 (41.07%) met the criteria for hypertension. Compared with normotensive individuals, the average duration of midday napping of subjects with hypertension was significantly longer (34.33 ± 0.58 min VS 31.46 ± 0.47 min, *p* < 0.001). Baseline characteristics of the longitudinal analysis categorized by midday nap duration are presented in Table 1. Among the 5729 participants included in the longitudinal analysis, 2839 (49.55%) individuals reported no napping, 495 (8.64%) participants had a midday nap less than 30 min, 485 (8.47%) subjects reported having a midday nap of 30–59 min, 1210 (21.12%) respondents reported a nap of 60–89 min/day and 700 (12.22%) had a midday nap equal to or longer than 90 min/day.

Table 2 below shows the associations between the midday nap duration and the risk of incident hypertension at four-year follow-up. The adjusted analysis shows that compared with participants who did not report napping, napping for ≥90 min/day was associated with significantly larger HR for hypertension (HR = 1.18, 95% CI = 1.01–1.40, *p* = 0.048).

We further conducted a stratified analysis by baseline characteristics. We observed a significantly larger risk of hypertension in individuals who reported both long sleep duration (≥8 h/night) and midday napping (≥90 min). We found that the association of hypertension with long midday nap duration (≥90 min/day) seemed to be more pronounced among participants who were overweight, or who either never smoke or drink, or without hyperlipidemia or diabetes. We also found that current-smokers who napped 1–30 min/day and ≥90 min/day were associated with a significantly larger risk of hypertension. In addition, the HR of developing hypertension at four-year follow-up for the older age subjects (≥60 years old) napped 60–89 min/day was 0.78 (Table A1).

Changes in midday nap duration from baseline (2011) and follow-up (2013) and the corresponding subsequent risk of hypertension in 2015 were presented in Table 3. Compared with people who napped ≥90 min/day both at baseline (2011) and follow-up (2013), after adjusting for confounders, we found significantly lower HR in people who decreased from ≥90 min/day at baseline (2011) to 1–59 min/day at follow-up (2013) (HR = 0.59, 95% CI = 0.36–0.97, *p* = 0.037) and to 60–89 min/day at follow-up (2013) (HR = 0.68, 95% CI = 0.47–0.99, *p* = 0.044).

## 4. Discussion

To our best knowledge, this is the first longitudinal cohort study to explore the association of midday nap duration and the change in midday nap duration with the risk of incident hypertension, using a nationwide representative sample of middle-aged and older Chinese adults. We found that, compared with participants without a habit of napping, long midday nap duration (≥90 min/day) was significantly associated with greater risks of hypertension at four-year follow-up. Compared with people who napped ≥90 min/day both at baseline (2011) and follow-up (2013), hypertension risks in 2015 was lower in middle-aged and older adults whose midday nap durations decreased in the 2-year study period from ≥90 min/day to 0–59 min/day and 60–89 min/day.

Previous studies have found that, relative to non-nappers, a higher prevalence of hypertension was found among people who frequently napped [18,19,20], and a meta-analysis conducted in 2016 found that the pooled RR (risk ratio) of hypertension in nappers was 1.13 [12], while some studies suggested that habitual napping was significantly associated with lower risk of hypertension [25,26]. Additionally, the associations between midday nap duration and hypertension were not consistent [21,22,23,24,27,28,29], especially in Chinese adults. Cai et al. found midday nap was significantly associated with a decreased prevalence of hypertension [27], and midday napping for more than 60 min was negatively associated with hypertension in Chinese adults living in rural areas [28,29]. However, a study including 13,469 Chinese adults over age 40 indicted, as compared to 0 min napping, all categories of daytime nap duration increased odds of incidence of hypertension [21]. Another cross-sectional study including 27,009 participants found that napping (≥30 min/day) was associated with a greater risk of hypertension among middle-aged and older Chinese [22]. In our analyses, among participants who napped 1–89 min/day, we found no significant association with hypertension, and lower HR for hypertension were observed in older age participants (≥60 years old) who napped 60–89 min/day. We also found longer midday nap duration (≥90 min/day) was associated with a significantly greater risk of hypertension, which is consistent with previous studies [22,23,24]. Additional large prospective studies are required to establish a clear relationship between midday nap duration and hypertension.

There are some plausible hypotheses that may help explain the relationship between midday napping and increased risk of hypertension. First, the morning has been recognized as the highest risk period of the day for cardiovascular events, with the surge in blood pressure caused by activation of the sympathetic nervous system [37]. Sympathetic surge also occurs when rising from a prolonged daytime nap resulting in rapid increases in blood pressure [38]. Second, midday napping could result in the elevation of evening cortisol levels [39], which may lead to high levels of blood pressure. Third, taking long napping may increases the total duration of sleep, and many studies have showed that longer sleep duration was associated with a greater risk of hypertension [40]. The findings of our study supported our speculation that we observed a significantly larger risk of hypertension in individuals who reported both long sleep duration (≥8 h/night) and midday napping (≥90 min). In addition, sleep apnea increases daytime and night-time blood pressure by neurogenic, hormonal, and vascular mechanisms and has been indicated as an important risk factor for hypertension [41]. Masa et al. demonstrated that people who with habitual midday nap having a greater frequency of sleep apnea [42], which may confound our observed relationships. Similarly, the association between midday nap and hypertension may be in part confounded by the indirect effect of midday nap on other diseases, such as diabetes and obesity. Several studies have indicated that midday nap is an important risk factor for diabetes and obesity in older adults [43,44]. Over two-thirds of type 2 diabetes patients were found with high blood pressure [45], and patients with obesity are more likely to report high blood pressure [46]. Notably, in our stratified analysis, longer midday nap duration (≥90 min/day) was found associated with significantly larger HR for hypertension in individuals with BMI ≥ 24, which was not observed among individuals with BMI < 24.

The current study, which utilized a retrospective cohort design, has the ability to investigate change in midday nap duration and subsequent risk of developing hypertension. We found that, compared with people who napped ≥90 min/day both at baseline (2011) and follow-up (2013), hypertension risk at four-year follow-up declined in middle-aged and older Chinese adults whose midday nap durations decreased in the 2-year study period from ≥90 min/day to 0–59 min/day and 60–89 min/day. Our findings suggest that people who had longer midday nap duration (≥90 min/day) but who decreased napping duration may experience a lower risk of developing hypertension. However, more research using objective measures of midday nap duration and blood pressure is required to further test the association.

The major strengths of our research lie in our utilization of a prospective cohort study to capture a nationally representative sample of middle-and older-age Chinese adults. This is the first cohort study that has evaluated the relationship between change in midday nap duration and hypertension. Besides, blood pressure was measured objectively and not self-reported in our research. Our study also has several limitations. First, measurement of midday nap duration and some health status indicators, such as smoking, drinking, depression, and personal medical histories, were based on self-reports and not objectively measured, which may lead to misclassification of exposure. Self-reported sleep duration estimated is usually longer than sleep duration from actigraphy or polysomnography [47]. Second, although we have adjusted for several factors that might confound the relationship between midday nap and hypertension, some confounders (such as salt intake, physical activity, sleep quality, obstructive sleep apnea) were not captured in our research [5,10,48,49]. Third, the survey in our study item asked specifically about napping after lunch, so we might miss some people who nap in the morning or evening. Fourth, this study was based on a population among middle-aged and older Chinese adults, our findings may not be applicable to populations in other countries.

## 5. Conclusions

This study provides evidence that longer midday nap duration (≥90 min/day) was significantly associated with larger risks of incident hypertension, and hypertension risk declined in whose midday napping decreased to 1–59 min/day. Considering that midday nap is a common modifiable behavior and hypertension is one of the strongest risk factors for common cardiovascular diseases worldwide, our findings offer information that can be used to make health-benefiting practical changes to prevent hypertension in the daily routines of individuals, especially in middle-aged and older Chinese adults. However, large, prospective longitudinal studies based on objective measures (such as actigraphy) of midday nap duration are needed to test the associations of midday nap duration and change in midday nap duration with hypertension among middle-aged and older Chinese adults. Additionally, the possible mechanisms behind the association need to be explored.

## Figures and Tables

**Table 1:** Baseline characteristics of participants by midday nap duration groups (N = 5729).

Variables	0 min (N = 2839)	1–29 min (N = 495)	30–59 min (N = 485)	60–89 min (N = 1210)	≥90 min (N = 700)	*p* Value ^3^
Age (years) ^1^	56.50 (13.00)	57.50 (12.00)	56.50 (12.00)	56.50 (14.00)	57.50 (15.00)	0.027
Gender ^2^						0.175
Male	1372 (48.33)	251 (50.71)	219 (45.15)	595 (49.17)	315 (45.00)	
Female	1467 (51.67)	244 (49.29)	266 (54.85)	615 (50.83)	385 (55.00)	
BMI (kg/m^2^) ^1^	22.32 (4.22)	22.92 (4.60)	22.49 (4.49)	22.84 (4.57)	22.74(4.76)	<0.001
Smoking status ^2^						<0.001
Current	815 (28.71)	158 (31.92)	130 (26.80)	421 (34.79)	276 (39.43)	
Former	162 (5.71)	40 (8.08)	33 (6.80)	126 (10.41)	67 (9.57)	
Never	1862 (65.59)	297 (60.00)	322 (66.39)	663 (54.79)	357 (51.00)	
Drinking status ^2^						<0.001
Current	828 (29.17)	178 (35.96)	158 (32.58)	473 (39.09)	289 (41.29)	
Former	171 (6.02)	25 (5.05)	38 (7.84)	84 (6.94)	55 (7.86)	
Never	1840 (64.81)	292 (58.99)	289 (59.59)	653 (53.97)	356 (50.86)	
SBP (mmHg) ^1^	118.33 (17.67)	119.33 (17.00)	118.00 (18.00)	117.67 (17.00)	119.00 (17.67)	0.229
DBP (mmHg) ^1^	70.33 (12.33)	71.33 (12.00)	70.67 (13.33)	69.67 (12.67)	71.00 (12.33)	0.039
Sleep duration (h) ^1^	6.00 (3.00)	7.00 (3.00)	6.00 (3.00)	7.00 (2.00)	7.00 (2.00)	<0.001
Depression ^2^	1380 (48.61)	220 (44.44)	233 (48.04)	540 (44.63)	317 (45.29)	0.091
Comorbidities ^2^						
Hyperlipidemia	109 (3.84)	31 (6.26)	27 (5.57)	62 (5.12)	48 (6.86)	0.004
Diabetes	80 (2.82)	13 (2.63)	26 (5.36)	49 (4.05)	28 (4.00)	0.019

^1^ Data are Median (IOR, interquartile range) for continuous variables (Shapiro-Wilk normality tests, *p* < 0.05). ^2^ Data are the frequency with percentage for categorical variables. ^3^ *p* Values were derived from analysis of Kruskal and Wallis tests for continuous variables and χ^2^ tests for category variables.

**Table 2:** Hypertension risk between 2011–2015 follow-up across categories of midday napping at baseline.

Midday Napping, Minutes	Cases/N	Person-Years	Unadjusted HR (95% CI) ^1^	*p* Value	Adjusted HR (95% CI) ^2^	*p* Value
0 min	600/2839	10,716	1.00 (ref.)		1.00 (ref.)	
1–29 min	121/495	1834	1.18 (0.97–1.43)	0.101	1.12 (0.92–1.36)	0.260
30–59 min	113/485	1818	1.11 (0.91–1.36)	0.309	1.10 (0.90–1.34)	0.366
60–89 min	244/1210	4592	0.95 (0.82–1.10)	0.492	0.89 (0.77–1.04)	0.133
≥90 min	185/700	2570	1.28 (1.09–1.51)	0.003	1.18 (1.01–1.40)	0.048

^1^ Pseudo R^2^ = 0.009. ^2^ Adjusted for age, gender, BMI, smoking status (current, former, or never), drinking status (current, former, or never), sleep duration, depression (yes or no), hyperlipidemia (yes or no) and diabetes (yes or no); Pseudo R^2^ = 0.036.

**Table 3:** Hypertension risk by subcategories of midday napping across baseline (2011) and follow-up (2013).

Midday Napping, Minutes		Cases/N	Person- Years	Unadjusted HR (95% CI)	*p* Value	Adjusted HR (95% CI) ^1^	*p* Value
2011	2013						
0 min	0 min	389/1752	6576	1.00 (ref.) ^2^		1.00 (ref.) ^6^	
	1–59 min	61/287	1080	0.95 (0.73–1.25)	0.738	0.95 (0.72–1.24)	0.701
	60–89 min	81/341	1274	1.07 (0.85–1.37)	0.556	1.00 (0.79–1.28)	0.973
	≥90 min	36/215	820	0.74 (0.53–1.05)	0.088	0.73 (0.52–1.03)	0.073
1–59 min	0 min	60/245	902	1.04 (0.73–1.47)	0.828	1.08 (0.76–1.53)	0.673
	1–59 min	68/285	1064	1.00 (ref.) ^3^		1.00 (ref.) ^7^	
	60–89 min	56/220	826	1.06 (0.75–1.51)	0.741	0.98 (0.69–1.40)	0.920
	≥90 min	39/140	506	1.20 (0.81–1.78)	0.36	1.13 (0.76–1.68)	0.551
60–89 min	0 min	56/256	962	1.11 (0.79–1.57)	0.544	1.18 (0.83–1.67)	0.359
	1–59 min	42/229	874	0.92 (0.63–1.34)	0.66	0.97 (0.66–1.42)	0.867
	60–89 min	75/380	1434	1.00 (ref.) ^4^		1.00 (ref.) ^8^	
	≥90 min	58/240	906	1.22 (0.87–1.72)	0.248	1.14 (0.80–1.61)	0.470
≥90 min	0 min	25/108	396	0.72 (0.46–1.12)	0.145	0.77 (0.49–1.20)	0.251
	1–59 min	19/102	384	0.57 (0.35–0.93)	0.025	0.59 (0.36–0.97)	0.037
	60–89 min	40/184	690	0.66 (0.46–0.96)	0.031	0.68 (0.47–0.99)	0.044
	≥90 min	89/281	1010	1.00 (ref.) ^5^		1.00 (ref.) ^9^	

^1^ Adjusted for age, gender, BMI, smoking status (current, former, or never), drinking status (current, former, or never), sleep duration, depression (yes or no), hyperlipidemia (yes or no) and diabetes (yes or no). ^2^ Pseudo R^2^ = 0.007; ^3^ Pseudo R^2^ = 0.010; ^4^ Pseudo R^2^ = 0.009; ^5^ Pseudo R^2^ = 0.007; ^6^ Pseudo R^2^ = 0.019; ^7^ Pseudo R^2^ = 0.031; ^8^ Pseudo R^2^ = 0.026; ^9^ Pseudo R^2^ = 0.021.

## Appendix A

**Table A1:** Hypertension risk and midday napping, stratified by baseline characteristics.

Variables	0 min (N = 2839)		1–29 min (N = 495)		30–59 min (N = 485)		60–89 min (N = 1210)		≥90 min (N = 700)	
	Person-Years	Adjusted HR	Person-Years	Adjusted HR (95% CI)	Person-Years	Adjusted HR (95% CI)	Person-Years	Adjusted HR (95% CI)	Person-Years	Adjusted HR (95% CI)
Age (years)										
45–60	7090	1.00 (ref.)	1144	1.23 (0.93–1.61)	1184	1.19 (0.90–1.57)	2900	1.01 (0.82–1.24)	1546	1.17 (0.92–1.50)
≥60	3626	1.00 (ref.)	690	1.01 (0.76–1.34)	634	1.00 (0.75–1.35)	1692	0.78 (0.62–0.97) *	1024	1.18 (0.94–1.48)
Gender										
Male	5184	1.00 (ref.)	940	1.14 (0.87–1.49)	808	1.19 (0.89–1.59)	2240	0.95 (0.77–1.17)	1158	1.19 (0.93–1.52)
Female	5532	1.00 (ref.)	894	1.09 (0.82–1.45)	1010	1.03 (0.78–1.36)	2352	0.85 (0.68–1.05)	1412	1.18 (0.94–1.48)
BMI (kg/m^2^)										
<24	7474	1.00 (ref.)	1170	1.09 (0.85–1.40)	1242	1.01 (0.78–1.31)	2966	0.85 (0.70–1.02)	1614	1.10 (0.89–1.37)
≥ 24	3242	1.00 (ref.)	664	1.17 (0.85–1.60)	576	1.25 (0.90–1.72)	1626	0.97 (0.76–1.24)	956	1.31 (1.01–1.71) *
Sleep duration (hours)										
≤6	5582	1.00 (ref.)	884	0.94 (0.70–1.26)	960	1.04 (0.79–1.38)	2134	0.86 (0.69–1.06)	1024	1.08 (0.83–1.40)
6–8	2034	1.00 (ref.)	408	1.32 (0.88–1.97)	376	0.90 (0.56–1.46)	1040	0.86 (0.62–1.20)	504	1.28 (0.89–1.83)
≥8	3100	1.00 (ref.)	542	1.32 (0.93–1.89)	482	1.34 (0.92–1.94)	1418	1.00 (0.76–1.32)	1042	1.35 (1.02–1.78) *
Smoking										
Current	3094	1.00 (ref.)	560	1.54 (1.12–2.11) **	484	1.18 (0.81–1.71)	1574	1.10 (0.86–1.41)	992	1.34 (1.02–1.75) *
Former	602	1.00 (ref.)	152	0.81 (0.38–1.75)	120	1.43 (0.73–2.83)	474	1.09 (0.69–1.73)	246	1.23 (0.70–2.14)
Never	7020	1.00 (ref.)	1122	1.15 (0.88–1.49)	1214	1.01 (0.76–1.33)	2544	0.92 (0.75–1.13)	1332	1.27 (1.01–1.60) *
Drinking										
Current	3116	1.00 (ref.)	660	1.22 (0.89–1.66)	590	1.15 (0.82–1.61)	1774	0.93 (0.73–1.18)	1050	1.14 (0.87–1.49)
Former	638	1.00 (ref.)	94	0.49 (0.15–1.60)	142	1.37 (0.73–2.57)	322	0.75 (0.42–1.34)	198	1.09 (0.59–1.98)
Never	6962	1.00 (ref.)	1080	1.16 (0.89–1.50)	1086	1.02 (0.77–1.34)	2496	0.91 (0.74–1.11)	1322	1.30 (1.03–1.64) *
Depression										
Yes	5220	1.00 (ref.)	808	1.11 (0.82–1.50)	890	0.96 (0.70–1.31)	2054	0.82 (0.65–1.04)	1166	1.18 (0.92–1.52)
No	5496	1.00 (ref.)	1026	1.13 (0.87–1.47)	928	1.18 (0.91–1.55)	2538	0.94 (0.78–1.15)	1404	1.17 (0.93–1.46)
Hyperlipidemia										
Yes	408	1.00 (ref.)	112	0.88 (0.35–2.23)	104	0.49 (0.14–1.67)	244	0.79 (0.37–1.68)	176	1.30 (0.64–2.61)
No	10,308	1.00 (ref.)	1722	1.14 (0.94–1.40)	1374	1.14 (0.93–1.39)	4592	0.91 (0.78–1.06)	3260	1.20 (1.01–1.42) *
Diabetes										
Yes	292	1.00 (ref.)	48	0.46 (0.11–1.95)	100	0.82 (0.35–1.91)	188	0.65 (0.33–1.30)	104	0.82 (0.35–1.94)
No	10,424	1.00 (ref.)	1786	1.15 (0.94–1.40)	1718	1.11 (0.90–1.37)	4404	0.90 (0.77–1.05)	2466	1.19 (1.00–1.41) *

Note: Adjusted for age, gender, BMI, smoking status (current, former, or never), drinking status (current, former, or never), sleep duration, depression (yes or no), hyperlipidemia (yes or no), and diabetes (yes or no). Each group adjusted for the other covariates. * *p* < 0.05, ** *p* < 0.01.
